# Identification of global regulators of T-helper cell lineage specification

**DOI:** 10.1186/s13073-015-0237-0

**Published:** 2015-11-20

**Authors:** Kartiek Kanduri, Subhash Tripathi, Antti Larjo, Henrik Mannerström, Ubaid Ullah, Riikka Lund, R. David Hawkins, Bing Ren, Harri Lähdesmäki, Riitta Lahesmaa

**Affiliations:** Turku Centre for Biotechnology, University of Turku and Åbo Akademi University, Turku, Finland; Department of Computer Science, Aalto University School of Science, Espoo, Finland; Division of Medical Genetics, Department of Medicine, University of Washington School of Medicine, Seattle, WA 98195 USA; Department of Genome Sciences, University of Washington School of Medicine, Seattle, WA 98195 USA; Ludwig Institute for Cancer Research, La Jolla, CA 92093 USA; Department of Cellular and Molecular Medicine, Institute of Genomic Medicine and Moores Cancer Center, University of California, San Diego, La Jolla, CA 92093 USA

## Abstract

**Background:**

Activation and differentiation of T-helper (Th) cells into Th1 and Th2 types is a complex process orchestrated by distinct gene activation programs engaging a number of genes. This process is crucial for a robust immune response and an imbalance might lead to disease states such as autoimmune diseases or allergy. Therefore, identification of genes involved in this process is paramount to further understand the pathogenesis of, and design interventions for, immune-mediated diseases.

**Methods:**

We aimed at identifying protein-coding genes and long non-coding RNAs (lncRNAs) involved in early differentiation of T-helper cells by transcriptome analysis of cord blood-derived naïve precursor, primary and polarized cells.

**Results:**

Here, we identified lineage-specific genes involved in early differentiation of Th1 and Th2 subsets by integrating transcriptional profiling data from multiple platforms. We have obtained a high confidence list of genes as well as a list of novel genes by employing more than one profiling platform. We show that the density of lineage-specific epigenetic marks is higher around lineage-specific genes than anywhere else in the genome. Based on next-generation sequencing data we identified lineage-specific lncRNAs involved in early Th1 and Th2 differentiation and predicted their expected functions through Gene Ontology analysis. We show that there is a positive trend in the expression of the closest lineage-specific lncRNA and gene pairs. We also found out that there is an enrichment of disease SNPs around a number of lncRNAs identified, suggesting that these lncRNAs might play a role in the etiology of autoimmune diseases.

**Conclusion:**

The results presented here show the involvement of several new actors in the early differentiation of T-helper cells and will be a valuable resource for better understanding of autoimmune processes.

**Electronic supplementary material:**

The online version of this article (doi:10.1186/s13073-015-0237-0) contains supplementary material, which is available to authorized users.

## Background

CD4+ T-helper (Th) cells are critical players in adaptive immune responses and protect the host against various pathogens. Naive CD4+ T cells are multi-potent in nature and have an ability to differentiate into distinct effector and regulatory subtypes that express lineage-specific regulators, including transcription factors and signature cytokines. For example, Th1 cells express the master transcription factor gene *TBX21* and secrete interferon γ and Th2 cells express *GATA3* and secrete interleukin (IL)4 and IL13 cytokines. Because these effector T-helper cell lineages are crucial for mounting distinct immune responses, inappropriate execution of their differentiation processes may result in imbalance between T-helper cell subsets and ultimately lead to various inflammatory autoimmune diseases and allergic responses [1–3]. To understand and develop potential therapeutic treatment regimes, it is important to get a high-resolution map of regulators involved in T-helper cell differentiation. Previous studies have identified elements involved in T-helper cell differentiation [4–8].

Lineage-specificity is a dynamic process that involves molecular mechanisms resulting in the expression of genes that establish lineage-specific gene expression and/or suppress alternative developmental fates. Transcriptional regulation is one way of achieving lineage-specificity. Only a small portion of RNA is translated into proteins, although vast chunks of human DNA are transcribed [9, 10]. These translated mRNAs are designated protein-coding genes. Epigenetic mechanisms represent the second layer of lineage-specific gene expression and involve histone modification, DNA methylation and non-coding RNAs [11–14]. We have previously shown that lineage-specific enhancer elements are at work in driving the expression of lineage-specific genes in Th1 and Th2 cells [15]. Long non-coding RNAs (lncRNA) are non-coding RNAs that are more than 200 nucleotides in length and do not have an open reading frame [16]. Recent studies show that non-coding RNAs that are not translated appear to be part of the vast regulatory machinery [17, 18].

In this study, we aimed at identifying lineage-specific mRNAs and lncRNAs participating in early differentiation (72 h) of Th1 and Th2 cells by comparing them to naïve (Thp) and activated CD4+ T cells (Th0). We utilized transcriptional profiling data from three different profiling platforms to get a high-confidence list of genes involved in T-helper cell lineage specification. By employing next-generation sequencing techniques, we were able to identify genes that were not previously known in the context of T-helper cell differentiation. Utilizing the same sequencing data, we were able to determine lineage-specific lncRNAs involved in early T-helper cell differentiation. We observed that there is a positive trend in the expression of lineage-specific lncRNAs lying in the vicinity of lineage-specific genes. In addition, using genome-wide data on histone modifications from Th1 and Th2 cells at 72 h, we also found that lineage-specific enhancers and promoters are more preferentially located around lineage-specific genes/lncRNAs than anywhere else in the genome. This shows the highly selective nature of the regulatory elements involved in T-helper cell differentiation. In addition, we further characterized lineage-specific lncRNAs for their predicted functions through Gene Ontology (GO) analysis using an lncRNA–mRNA co-expression network. This will be a valuable resource for further studies since the function of majority of lncRNAs is unknown.

## Methods

### Ethics statement

This study was approved by the Ethics Committee of the Hospital district of Southwest Finland in line with the 1975 Declaration of Helsinki. Informed consent was obtained from each donor.

### Human cord blood CD4+ T-cell isolation and culturing

Naive CD4+ T cells were isolated from human umbilical cord blood of healthy neonates born in Turku University Central Hospital. Mononuclear cells were isolated using Ficoll-Paque gradient centrifugation (Amersham Pharmacia Biotech, Uppsala, Sweden) and CD4+ T cells were purified using positive selection (Dynal CD4 Positive Isolation Kit, Invitrogen, Carlsbad, CA, USA). CD4+ T cells from several individuals were pooled after the isolation. Purified CD4+ T cells were cultured in Yssel’s medium (Iscove’s modified Dulbecco’s medium supplemented with Yssel medium concentrate plus penicillin/streptomycin) supplemented with 1 % human AB serum (Red Cross Finland Blood Service). Cells were activated with plate-bound anti-CD3 (2.5 μg/ml) and soluble anti-CD28 (500 ng/ml; both were from Immunotech, Marseille, France). Simultaneously, Th1 polarization was initiated with 2.5 ng/ml IL12 and Th2 neutralizing antibody anti-IL4 (1 μg/ml); Th2 differentiation was promoted using 10 ng/ml IL4 plus Th1 neutralizing antibody anti-interferon γ (1 μg/ml) (all antibodies from R&D Systems, Minneapolis, MN, USA); or cells were cultured with only neutralizing antibodies (anti-interferon γ and anti-IL4) and without polarizing cytokines (Th0 cells). IL2 (40 U/ml, R&D Systems) was added on the second day of culture. Further, cells were supplemented with media and divided every second day to keep the polarizing conditions during the culture until day 7. The polarization was verified by checking the expression of polarization marker genes for Th1 and Th2 subsets.

### RNA isolation and transcriptional profiling

Total RNA was extracted from naïve precursor human cord blood CD4+ T cells, activated Th0 cells, and differentiated Th1 and Th2 cells at 72 h using Trizol reagent (Invitrogen). For hybridization on the Affymetrix Human Genome U133 Plus 2.0 array, 250 ng of total RNA was used as starting material and was processed with an Affymetrix GeneChip 3’ IVT Express kit according to the sample preparation guide. For hybridization on the Illumina HumanHT-12 v4 Expression BeadChip, 300 ng of total RNA was used as starting material and was processed with an Illumina TotalPrep RNA Amplification kit according to the sample preparation guide. For sequencing, 400 ng of total RNA was used as starting material and libraries were prepared with an Illumina TrueSeq RNA Sample Prep kit v2 according to the sample preparation guide. The sequencing data were generated using an Illumina HiSeq-2000 instrument and the number of reads obtained can be found in Additional file 1. These transcriptional profiling data have been deposited in Gene Expression Omnibus (GEO) under accession [GEO:GSE71646].

### Analysis of Affymetrix microarray data

The R statistical environment was used for analysis. Affymetrix microarray data were normalized using the robust multi-array average algorithm implemented in the affy package [17]. Duplicate and un-annotated probes were removed using the genefilter package [19]. The probeset with the highest inter-quartile range was retained in case of duplicates. Present and absent calls for probesets were generated by fitting the chip-wide log2-transformed expression data to a two-component Gaussian distribution function, using the standard Expectation-Maximization (EM) algorithm implemented in the mixtools package [20]. A probeset was defined to be present if the corresponding data point had a higher likelihood for the Gaussian component with a higher mean value in all the replicates of the sample subtype [21]. Differential expression analysis was done using moderated unpaired t-test as implemented in limma [22]. The genes were considered as differentially expressed if the Benjamini-Hochberg adjusted *p* value < 0.05 and log2 fold-change < −1 or > 1.

### Analysis of Illumina microarray data

The R statistical environment was used for analysis. Illumina microarray data were preprocessed, including background adjustment, variance stabilization transformation and quantile normalization as implemented in the lumi package [23]. Duplicate and un-annotated probes were removed using the genefilter package [19]. The probeset with the highest inter-quartile range was retained in case of duplicate probesets. Present and absent calls were obtained using the detection *p* value. A probeset was defined to be present if the detection *p* value < 0.01 in all the replicates of a sample subtype. Differential expression analysis was performed as described in analysis of Affymetrix microarray data.

### Analysis of RNA-sequencing data for gene expression

The quality of sequenced reads was checked using FastQC [24] and the reads were mapped to the hg19 reference transcriptome and genome build using TopHat [25]. Gene counts were obtained using the htseq-count script included in the htseq tool. Raw counts were normalized and variance-stabilized values were obtained using methods implemented in the DESeq package [26] in R. Present and absent calls were generated by fitting the normalized values to a two-component Gaussian distribution function using the EM algorithm implemented in the mixtools package in R [20]. A gene was defined to be present if the corresponding data point had a higher likelihood for the Gaussian component with a higher mean value in all the replicates of the sample subtype. Differential expression analysis was done on raw counts using the default settings in the DESeq package. The genes were considered to be differentially expressed if the Benjamini-Hochberg adjusted *p* value < 0.05 and modified log2 fold-change < −1 or > 1. The resulting genes were refined using the previously generated present and absent calls.

### Analysis of RNA-sequencing data to identify lncRNAs

Using the reads mapped to the hg19 reference genome, we estimated the expression levels of lncRNAs using the htseq-count script included in the htseq tool by providing the genomic features from the GENCODE v16 catalog of lncRNAs [27] along with the transcriptome. Differential expression of lncRNAs was done on raw counts using the default settings in the DESeq package [26]. The lncRNAs were considered to be differentially expressed if the Benjamini-Hochberg adjusted *p* value < 0.05 and modified log2 fold-change < −1 or > 1. We define a lineage-specific lncRNA to be in the vicinity of a lineage-specific gene if it is within 5 kb upstream or 30 kb downstream of the gene.

### Lineage-specific genes or lncRNAs

We selected all the genes that are differentially expressed in Thp versus Th0, Th1 and Th2 subsets from the three platforms and made a confident list of differentially expressed genes by checking that each gene was differentially expressed in at least two or more platforms with the same directionality in their fold change. In cases of novel genes or lncRNAs, we used the above comparisons from next-generation sequencing data only. We defined a feature to be Th1- or Th2-specific if it is uniquely differentially expressed in only Thp versus Th1 or Thp versus Th2 comparisons, respectively, but not differentially expressed in Thp versus Th0.

### Th1- and Th2-specific enhancer and promoter marks around lineage-specific genes/lncRNAs

We overlaid enhancer marks found in Th1 and Th2 cells from a previously published study [15] on lineage-specific genes/lncRNAs obtained in this study. We define an enhancer mark to be in the vicinity of a lineage-specific feature if it is within 125 kb upstream or downstream of the transcription start site of the feature. We also overlaid promoter marks found in Th1 and Th2 cells obtained from the same dataset on lineage-specific genes/lncRNAs. We define a promoter mark to be in the vicinity of a lineage-specific feature if it is within 2.5 kb upstream or downstream of the transcription start site of the feature. For randomization tests, we randomly (n = 10,000) picked the same number of genes as that of a lineage-specific set from anywhere else in the genome and quantified the overlap of enhancer and promoter marks around them. The *p* values were computed with respect to this randomly generated null distribution.

### Prediction of GO terms for lncRNAs

In order to predict GO terms for lncRNAs, we constructed a co-expression network of lncRNAs and protein-coding genes. We defined a lncRNA to be co-expressed with a protein-coding gene if the absolute Pearson’s correlation coefficient between their expression is greater than 0.9. For each group of protein-coding genes which are co-expressed with a particular lncRNA gene, we performed a topology based GO enrichment test as implemented in the topGO package in R [28]. Specifically, we used Fisher’s exact test and then attributed the enriched GO terms with a *p* value of < 0.01 to that specific lncRNA.

### Disease-associated single nucleotide polymorphism analysis

Disease-associated single nucleotide polymorphism (SNP) data were obtained from the National Center for Biotechnology Information (http://www.ncbi.nlm.nih.gov/projects/gapplusprev/sgap_plus.htm). All SNPs with a *p* value > 1e-5 were excluded from further analysis. A gene was defined to be associated with a SNP if it is within ±100 kb of the SNP. Enrichment analysis of traits was performed using hypergeometric distribution.

## Results

### Transcriptional analysis of Th1- and Th2-specific genes

**Fig. 1:**
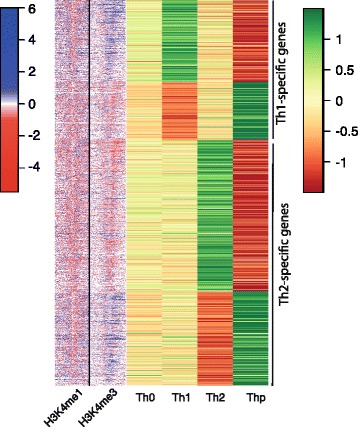
Th1- and Th2- specific genes and their associated epigenetic marks in the human genome. Heatmap showing gene expression and epigenetic profiles of Th1- and Th2-specific genes in T-helper subsets. The figure shows both high confidence and novel genes. Rows were first ordered based on log2 fold change and then by expression value. Normalized expression from sequencing data was standardized using Z-score for visualization purposes. In the case of H3K4me1 and H3K4me3 marks, the ratio of tag counts at the transcription start site between Th1 and Th2 is shown

Cellular differentiation to a specific subset requires activation of cell type-specific genes and suppression of genes of alternative lineages. To identify the lineage-specific genes, we analyzed transcriptional data for differential gene expression for Thp versus Th0, Th1 and Th2 subsets (Additional file 2). The number of genes determined to be present and available for analysis was 11,753 for Affymetrix arrays, 9210 for Illumina arrays and 13,744 for Illumina Sequencing (Figure S2a in Additional file 3). The transcriptomic platform comparison results are provided in Figure S2b, c in Additional file 3, and in Additional files 4, 5, and 6. According to our definition of lineage specificity and based on the data from the three platforms, there are 249 Th1-specifying genes and 491 Th2-specifying genes (Fig. 1; Additional file 7). These are confident lists of lineage-specific genes and have been internally validated as they are obtained from multiple sources. We also obtained a novel list of lineage-specific genes using next-generation sequencing data, in which there are 189 Th1-specific genes and 272 Th2-specific genes (Additional file 8). Among the lineage-specific genes, our analysis identified those encoding cytokines, chemokines, chemokine receptors, enzymes and transcription factors. Additionally, we found a panel of genes that were up-regulated and down-regulated in a lineage-specific manner. The Th1-specific ones include genes with both known and novel roles in Th1 cell differentiation. For example, *GIMAP4*, *CCL3*, *CXCR5*, *FUT7*, *IL21*, *TBKBP1*, *ABHD5* and *APOBEC3G* were up-regulated and *BACH2*, *CSTL*, *AFF3*, *TGFB3* and MAL were down-regulated specifically in the Th1 cell lineage. FUT7, an enzyme that catalyzes synthesis of sialyl Lewis^X^ antigens, has been shown to be expressed in CD4+ T cells [29]. Additionally it has binding sites for both GATA-3 and T-bet, master transcription factors for Th1 and Th2 cells where T-bet induces and GATA-3 inhibits the transcription of the *FUT7* gene [30]. CCL3 (MIP-1α) has previously been shown to be associated with the type 1 immune response [31]. CXCR5 is a chemokine receptor expressed on follicular T-helper cells. *APOBEC3G* expression is regulated in different CD4+ T-helper cells and is critical for modulation of HIV infectivity [32, 33]. TBKBP1 is involved in TNF-α–NF-kB interaction and potentially has a critical role in antiviral innate immunity [34]. Genes down-regulated in response to Th1 differentiation and whose expression is increased in alternative lineages include *CSTL*, *AFF3*, and *TGFB3*, which are expressed in Th17 cells [35], and *BACH2* and *MAL*, which are expressed in Th2 cells [36]. Th2 marker genes include those encoding the transcription factors GATA3 and GFI1 and lineage-specific cytokines, e.g., IL13, CCL17, and CCL20 [37–40]. Other genes include *THY1*, *NOD2*, *SOCS1*, *ABHD6*, *PPP1R14A*, *PPARG*, and *BCAR3*. The role of THY1 and NOD2 has been documented in Th2 differentiation [41–43]. However, the role of *ABHD6*, *PPP1R14A*, *PPARG*, and *BCAR3* in Th2 development remains to be determined.

**Fig. 2:**
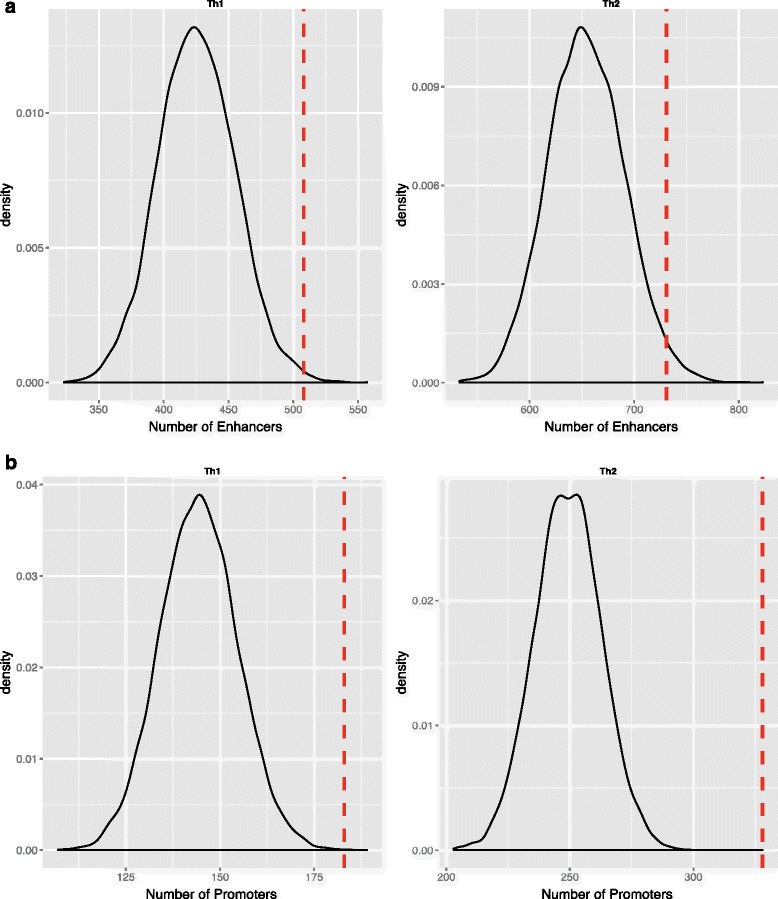
Randomization test performed to check the density of lineage-specific epigenetic marks across the genome. **a** Randomization test reveals that the number of lineage-specific enhancers around lineage-specific genes is more than anywhere else in the genome. **b** Randomization test reveals that the number of lineage-specific promoters around the lineage-specific genes is more than anywhere else in the genome. These results indicate the specific nature of regulatory elements involved in early T-helper cell differentiation. The distribution of enhancers or promoters in the vicinity of genes in the genome was determined by randomly picking the same number of genes as in the lineage-specific set. The *red dashed line* indicates the number of lineage-specific enhancers or promoters found in the vicinity of lineage-specific genes

We further validated the lineage specificity of these genes using lineage-specific enhancers and promoters. Enhancers and promoters were previously found to be differentially methylated at lysine 4 of histone H3 proteins [44]. We expected to find more active enhancer and promoter marks around lineage-specific genes than anywhere else in the genome. In order to determine the lineage-specific enhancers around lineage-specific genes, we overlaid lineage-specific enhancers from a previous study [15]. We found 508 Th1 enhancers around Th1-specific genes and 731 Th2 enhancers around Th2-specific genes (Fig. 2a). We then performed a randomization experiment (10,000 times) to compare the density of lineage-specific enhancers with that anywhere else in the genome. We found that there are more lineage-specific enhancers around lineage-specific genes than anywhere else in the genome (Th1 *p* value = 0.0038; Th2 *p* value = 0.0196; Fig. 2a). We repeated the same procedure with active promoters and found out that there are 183 Th1 active promoters, defined by the presence of both H3K4me3 and H3K27ac marks, around Th1-specific genes and 328 Th2 active promoters around Th2-specific genes. Randomization test results showed that there are more lineage-specific active promoters around lineage-specific genes than anywhere else in the genome (Th1 *p* value = 0.0003; Th2 *p* value < 10^−4^). These findings suggest the specific nature of genes and their epigenetic marks in T-helper cell differentiation.

We also looked for overlap between disease-associated SNPs and lineage-specific genes found in this study to explore their role in immune-mediated diseases. SNPs belonging to immune-mediated diseases, including asthma and Hodgkin disease, were found to be enriched in Th2-specific genes. Additionally, we found that SNPs belonging to other diseases were also enriched in Th1- and Th2-specific genes (Table 1).

**Table 1:** Enrichment of disease-associated SNPs in Th1- and Th2-specific genes

Disease	*P* value
Th1-specific genes	
Endometriosis	0.0016
Ovarian neoplasms	0.0087
Narcolepsy	0.0311
Th2-specific genes	
Hodgkin disease	0.0119
Moyamoya disease	0.0256
Osteoarthritis	0.0256
Asthma	0.0259
Osteoarthritis, knee	0.0393
Diabetes mellitus, type 2	0.0481

### Identification of lineage-specific lncRNAs in Th1 and Th2 subsets

**Fig. 3:**
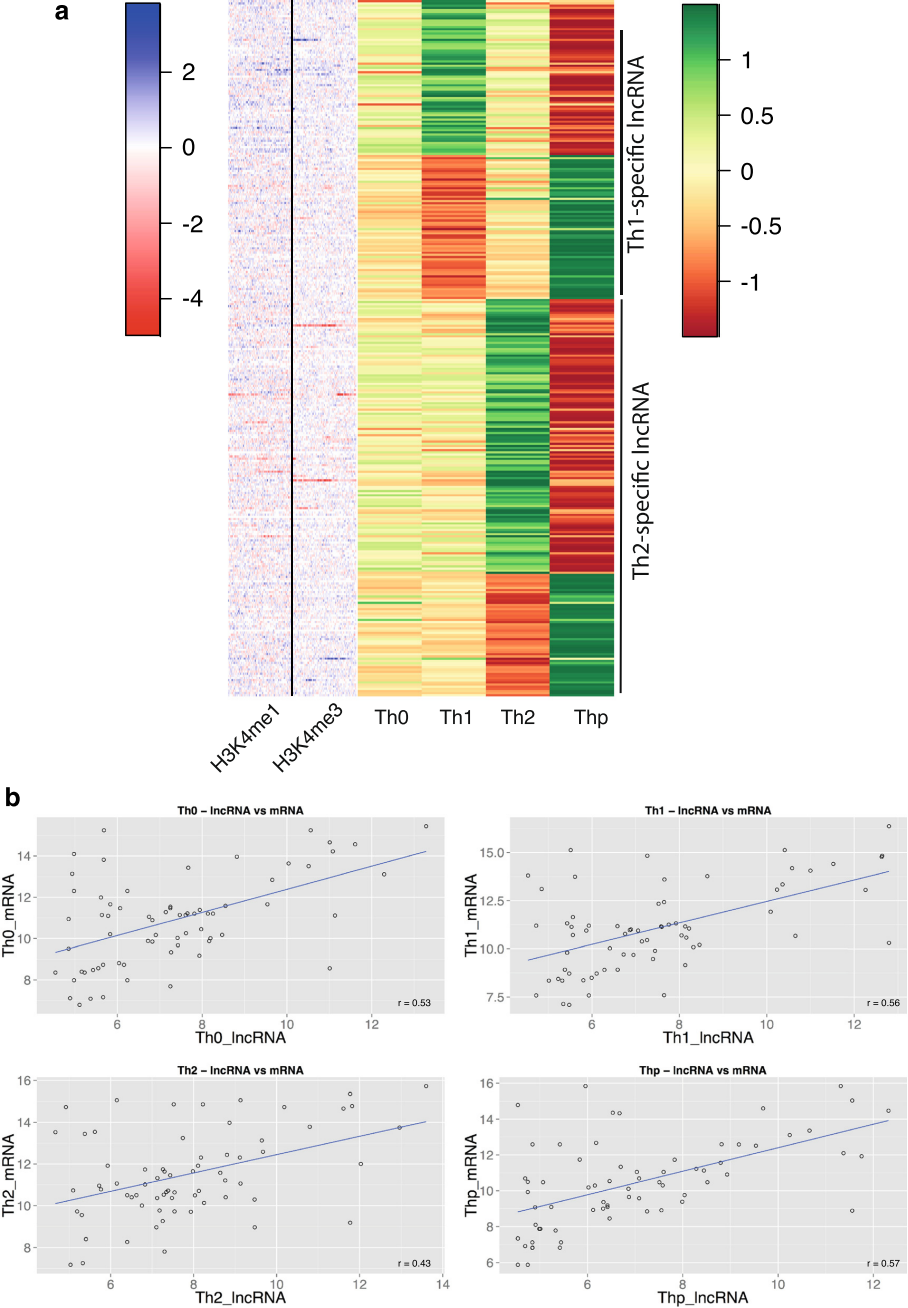
lncRNAs involved in early T-helper cell differentiation. **a** Heatmap showing expression and epigenetic profiles of Th1- and Th2-specific lncRNAs in T-helper cell subsets. Rows were first ordered based on log2 fold change and then by expression value. Normalized expression data from sequencing data were standardized using Z-score for visualization purposes. In the case of H3K4me1 and H3K4me3 marks, the ratio of tag counts at the transcription start site between Th1 and Th2 is shown. **b** Correlation plots of lineage-specific lncRNAs and lineage-specific genes in various T-helper cell subsets

In order to find lineage-specific lncRNAs, we determined differentially expressed lncRNAs between Thp versus Th0, Th1 and Th2 subsets. By our definition of lineage specificity, there are 136 Th1 lineage-specific lncRNAs and 181 Th2 lineage-specific lncRNAs (Fig. 3a; Additional file 9). These lineage-specific lncRNAs can be classified into antisense (152), intergenic (83), processed transcript (62), sense intronic (15), sense overlapping (4) and 3’ overlapping (1) based on their location in the genome. In accordance with previous studies [45], we observed that lncRNAs have lower expression than protein coding genes (Additional file 10). However, lineage-specific lncRNAs are expressed at a higher level than the rest of the lncRNAs (Additional file 10) as reported in a recent study [46]. We then looked for lineage-specific lncRNAs that are in the vicinity of lineage-specific genes. There are 24 Th1 lineage-specific lncRNAs around Th1 lineage-specific genes and 47 Th2 lineage-specific lncRNAs around Th2-specific genes (Additional file 11). We observed a positive trend between the expression of these lineage-specific lncRNAs and lineage-specific genes (Fig. 3b).

We also looked at the relationship between lineage-specific lncRNAs and epigenetic marks that lie in their vicinity. We followed the same approach as that used for lineage-specific genes to determine enhancers and the epigenetic state of promoters around lineage-specific lncRNAs; 392 Th1 enhancers and 53 Th1 promoters were found in the vicinity of Th1-specific lncRNAs and 372 Th2 enhancers and 61 Th2 promoters were found in the vicinity of Th2-specific lncRNAs. Interestingly, the H3K4me1 and H3K4me3 histone mark maps in Fig. 3a do not show such an obvious pattern associated with differential gene expression as seen for lineage-specific coding genes (Fig. 1). However, randomization tests revealed that the number of lineage-specific enhancers and promoters around lineage-specific lncRNAs are highly enriched compared with anywhere else in the genome (Figure S5a, b in Additional file 12). We then looked for overlap between disease-associated SNPs and lineage-specific lncRNAs and found many disease-associated SNPs (including immune-mediated diseases) that are enriched in the vicinity of Th1- and Th2-specific lncRNAs, suggesting they have a role in these diseases (Table 2).

**Table 2:** Enrichment of disease-associated SNPs in Th1- and Th2-specific lncRNAs

Trait	*P* value
Th1-specific lncRNAs	
Biliary atresia	0.007
Hepatitis C	0.008
Breast neoplasms	0.009
Diabetes mellitus, type 1	0.015
Cleft palate	0.018
Ovarian neoplasms	0.023
Diabetes mellitus, type 2	0.026
Leukemia, lymphoid	0.026
Diabetic nephropathies	0.029
Hypothyroidism	0.042
Th2-specific lncRNAs	
Cleft lip	0.001
Lupus erythematosus, systemic	0.002
Parkinson disease	0.003
Stroke	0.003
Inflammatory bowel diseases	0.003
Diabetes mellitus, type 2	0.005
Biliary atresia	0.009
Osteoarthritis	0.009
Colitis, ulcerative	0.01
Breast neoplasms	0.015
Thyroid neoplasms	0.019
Esophagitis	0.022
Gallbladder diseases	0.022
Arthritis, rheumatoid	0.027
Supranuclear palsy, progressive	0.028
Alzheimer disease	0.032
Rhinitis, allergic, seasonal	0.043
Coronary artery disease	0.046
Colorectal neoplasms	0.048

### Functional characterization of identified lncRNAs

Very little is known about the function of lncRNAs, but as shown in previous studies [47], co-expressed genes participate in similar functions. Therefore, we constructed a co-expression network of lncRNAs and protein-coding genes. We then looked for GO terms enriched among the co-expressed genes and attributed the enriched GO terms to the lncRNAs. The GO terms enriched in lineage-specific lncRNAs are summarized in Additional file 13 and a complete list can be found in Additional file 14. These GO terms aid in understanding the role of these lncRNAs in various biological processes.

## Discussion

T-helper cell differentiation is a complex process and some previous studies have elucidated the genes involved in it [4–8]. Since most of the previous studies have used microarrays for global profiling of the transcriptome, they are limited by factors such as pre-selection bias and probe design [48]. In our study, we use multiple transcriptional profiling platforms to generate a high-confidence list of genes that are involved in T-helper cell speciation. In addition, we complement the high-confidence list of genes with a novel list of genes inferred from only next-generation sequencing data. This novel list has many genes that are not previously known in the context of T-helper cell differentiation.

In the process of obtaining these lineage-specific genes, we also compared the transcriptomic profiling platforms used. Our platform comparison results are in concordance with previously published studies [49, 50]. The detection range of Illumina arrays is narrow compared with that of Affymetrix arrays and Illumina sequencing. These results aid in future experimental design, e.g., a next-generation sequencing platform is a good choice when one intends to study low-abundance genes.

To distinguish genuine expression from background noise, we generated present/absent calls for genes for each platform. In the case of Illumina arrays, well-defined negative probes enabled easy estimation of background and generation of detection *p* values. In the case of Affymetrix arrays, the negative probes did not have a desirable behavior. Therefore, we have used Gaussian mixture modeling to estimate the probability of a gene being genuinely expressed. In the case of Illumina sequencing data, we used normalized data obtained after variance stabilization in estimating genuinely expressed genes using Gaussian mixture modeling.

Since next-generation sequencing data can be leveraged to quantify other transcripts, such as lncRNAs, we determined lineage-specific lncRNAs. Previous studies [46, 51] identified lncRNAs in completely differentiated T-helper cells but, to our knowledge, this is the first study with global profiles of lncRNAs involved in early stages of human Th1 and Th2 cell differentiation. Additionally, our analysis revealed the relationship between lineage-specific lncRNAs and lineage-specific gene expression and found that the lineage-specific lncRNAs and lineage-specific gene expression are positively correlated. The finding led us to speculate that some of the lncRNAs might be acting as either enhancer elements during T-helper cell differentiation as suggested by a previous study [9] or that the lncRNA and gene pair can be regulated by another factor as suggested by Hu et al. [51].

We also quantified the enrichment of disease SNPs in the vicinity of lineage-specific genes and lncRNAs. SNPs associated with both immune-mediated and non-immune-mediated diseases were enriched around Th1- and Th2-specific genes and lncRNAs. This suggests that besides immune-mediated ones, these elements might also be involved in other cellular processes. With recent advancements in genome-editing technologies like CRISPR/Cas9, it will be possible to determine how a given SNP in a regulatory region might influence cellular functions involved in disease pathogenesis.

## Conclusion

The results show the involvement of several new actors in the early differentiation of T-helper cells and the relationship between epigenetic factors and lncRNAs and their possible role in autoimmune diseases.

## Additional files

Additional file 1: Table S1. Reads obtained from next-generation sequencing data. (XLSX 43 kb) Additional file 2: Figure S1. Analysis design schematic. (PDF 133 kb) Additional file 3: Figure S2. Comparison of transcriptional profiling platforms. a Genes determined to be present in T-helper cell subsets in the three platforms used for transcriptional profiling. b Gene expression density curves of T-helper cell subsets in the three platforms profiled. Only genes determined to be present were included. c Box plot of expression of genes in T-helper cell subsets based on their detection in the platforms used for transcriptional profiling. (PDF 117 kb) Additional file 4: Figure S3. Distribution of lineage-specific genes in platforms when analyzed individually in each platform. (PDF 103 kb) Additional file 5: Table S2. Table showing Jaccard similarity coefficients between platforms (XLSX 34 kb) Additional file 6: Table S3. Table showing Pearson correlation coefficients between platforms. (XLSX 34 kb) Additional file 7: Table S4. High confidence list of Th1- and Th2-specific genes in human. (XLSX 399 kb) Additional file 8: Table S5. Novel list of Th1- and Th2-specific genes in human. (XLSX 122 kb) Additional file 9: Table S6. List of Th1- and Th2-specific long non-coding RNAs. (XLSX 97 kb) Additional file 10: Figure S4. Relative abundance of various lncRNA types along with protein-coding genes. The column names are as follows: *AS* anti sense lncRNA, *AS_L* lineage-specifc antisense lncRNA, *linc* long intergenic non-coding RNA, *linc_L* lineage-specific lincRNA, *PT* processed transcript, *PT_L* lineage-specific processed transcript, *SI* sense intronic, *SI_L* lineage-specific sense intronic, *SO* sense overlapping, *SO_L* lineage-specific sense overlapping, *PC* protein-coding mRNA. (PDF 132 kb) Additional file 11: Table S7. List of lineage-specific lncRNAs in the vicinity of lineage-specific protein coding genes. (XLSX 42 kb) Additional file 12: Figure S5. Randomization test of epigenetic marks around lineage-specific lncRNAs. a Randomization test reveals that the number of lineage-specific enhancers around lineage-specific lncRNAs is more than anywhere else in the genome. b Randomization test reveals that the number of lineage-specific promoters around the lineage-specific lncRNAs is more than anywhere else in the genome. The distribution of enhancers or promoters in the vicinity of genes in the genome was determined by randomly picking the same number of genes as in the lineage-specific set. The *red dashed line* indicates the number of lineage-specific enhancers or promoters found in the vicinity of lineage-specific lncRNAs. (PDF 114 kb) Additional file 13: Table S8. GO terms enriched in Th1- and Th2- specific lncRNAs. (XLSX 99 kb) Additional file 14: Table S9. Predicted GO terms of lineage-specific lncRNAs. (XLSX 380 kb)

## Abbreviations

EM: Expectation-Maximization
GO: Gene Ontology
IL: interleukin
lncRNA: long non-coding RNA
SNP: single nucleotide polymorphism
Th: T-helper
