# Supplementary figures and images for: Identification of global regulators of T-helper cell lineage specification
Source: Genome Med. 2015 Nov 20;7:122. doi: 10.1186/s13073-015-0237-0 (PMC4654807; doi:10.1186/s13073-015-0237-0)

SI Fig 1

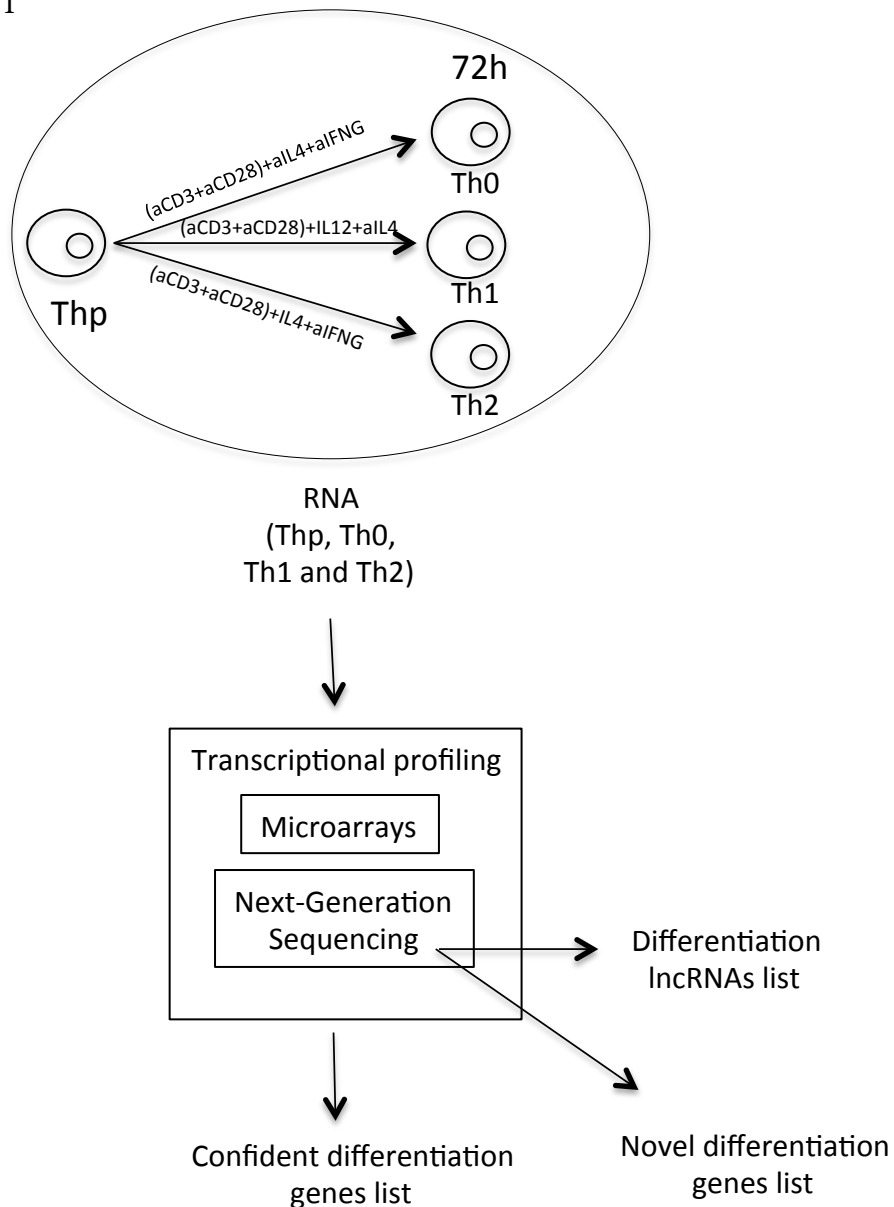

Supplement: Additional file 2: Figure S1. — Analysis design schematic. (PDF 133 kb) [file 13073_2015_237_MOESM2_ESM.pdf]

SI Fig 2

A

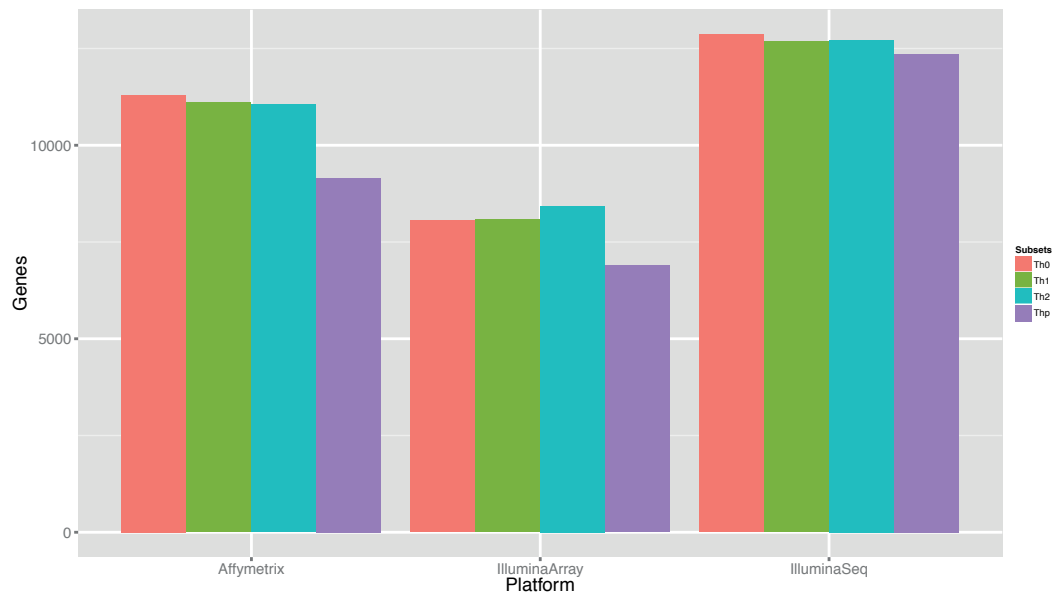

B

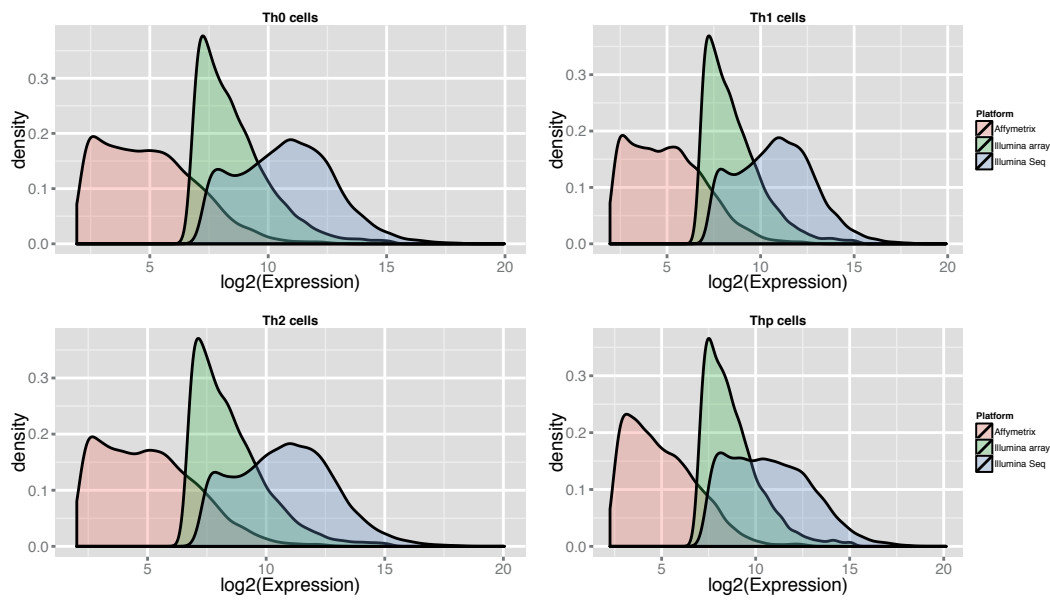

C

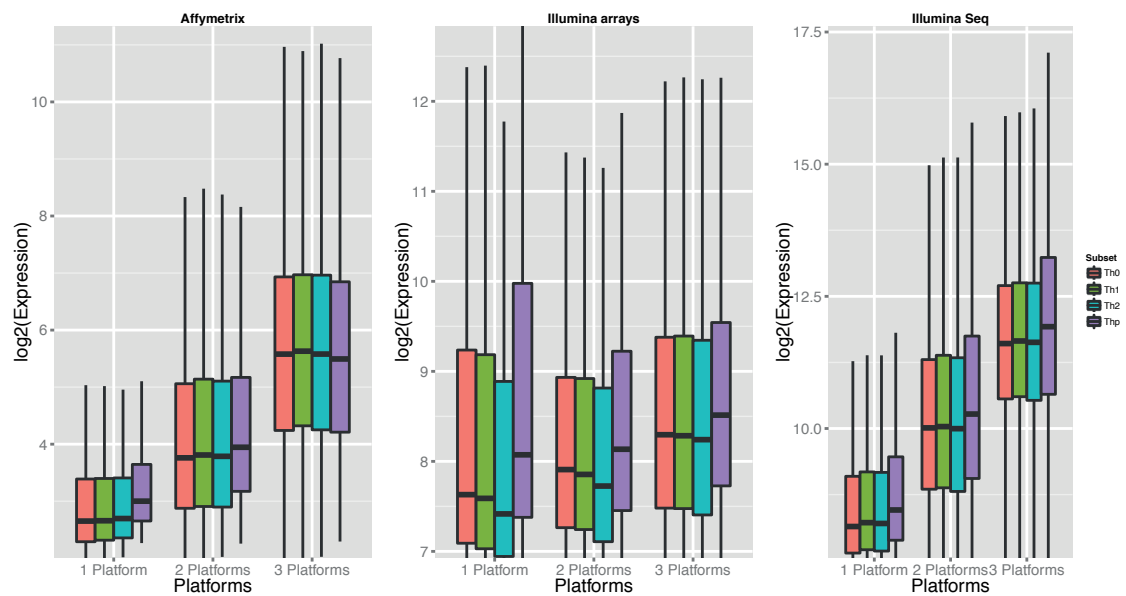

Supplement: Additional file 3: Figure S2. — Comparison of transcriptional profiling platforms. a Genes determined to be present in T-helper cell subsets in the three platforms used for transcriptional profiling. b Gene expression density curves of T-helper cell subsets in the three platforms profiled. Only genes determined to be present were included. c Box plot of expression of genes in T-helper cell subsets based on their detection in the platforms used for transcriptional profiling. (PDF 117 kb) [file 13073_2015_237_MOESM3_ESM.pdf]

SI Fig - 3

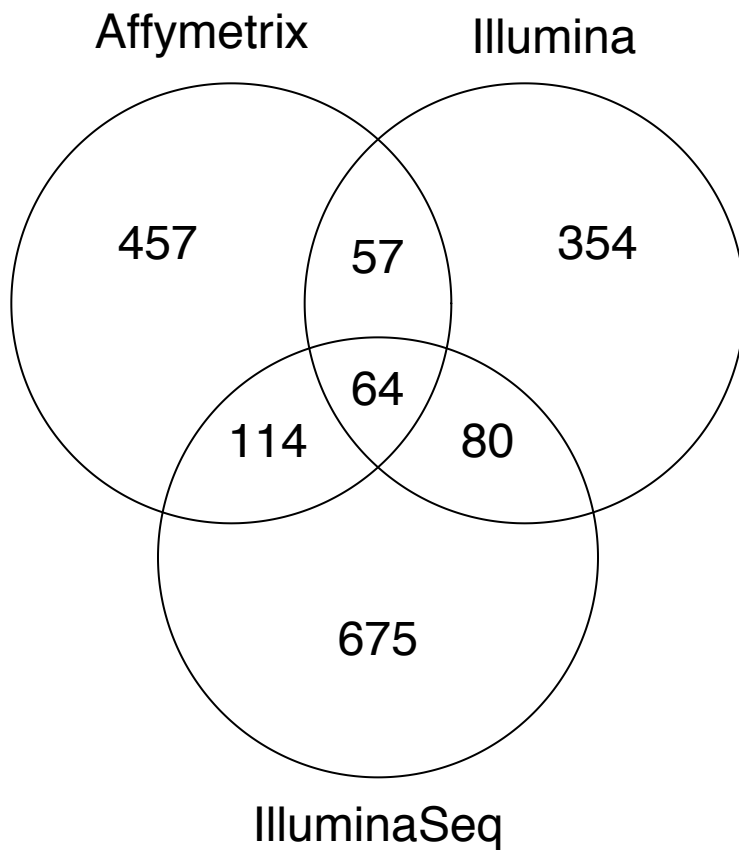

Distribution of lineage-specific genes in platforms when analysed individually

Supplement: Additional file 4: Figure S3. — Distribution of lineage-specific genes in platforms when analyzed individually in each platform. (PDF 103 kb) [file 13073_2015_237_MOESM4_ESM.pdf]

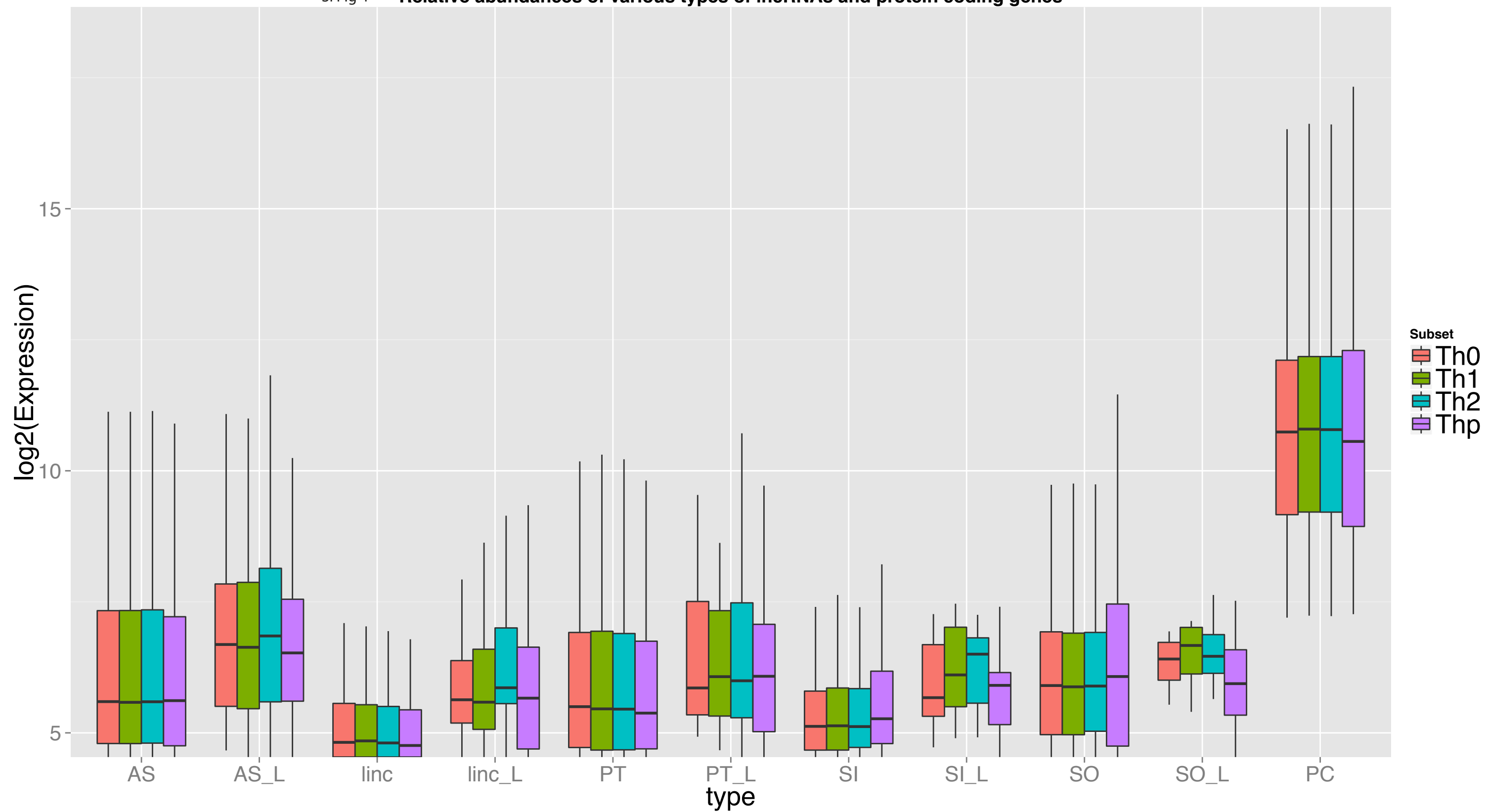

Supplement: Additional file 10: Figure S4. — Relative abundance of various lncRNA types along with protein-coding genes. The column names are as follows: AS anti sense lncRNA, AS_L lineage-specifc antisense lncRNA, linc long intergenic non-coding RNA, linc_L lineage-specific lincRNA, PT processed transcript, PT_L lineage-specific processed transcript, SI sense intronic, SI_L lineage-specific sense intronic, SO sense overlapping, SO_L lineage-specific sense overlapping, PC protein-coding mRNA. (PDF 132 kb) [file 13073_2015_237_MOESM10_ESM.pdf]

SI Fig 5

A

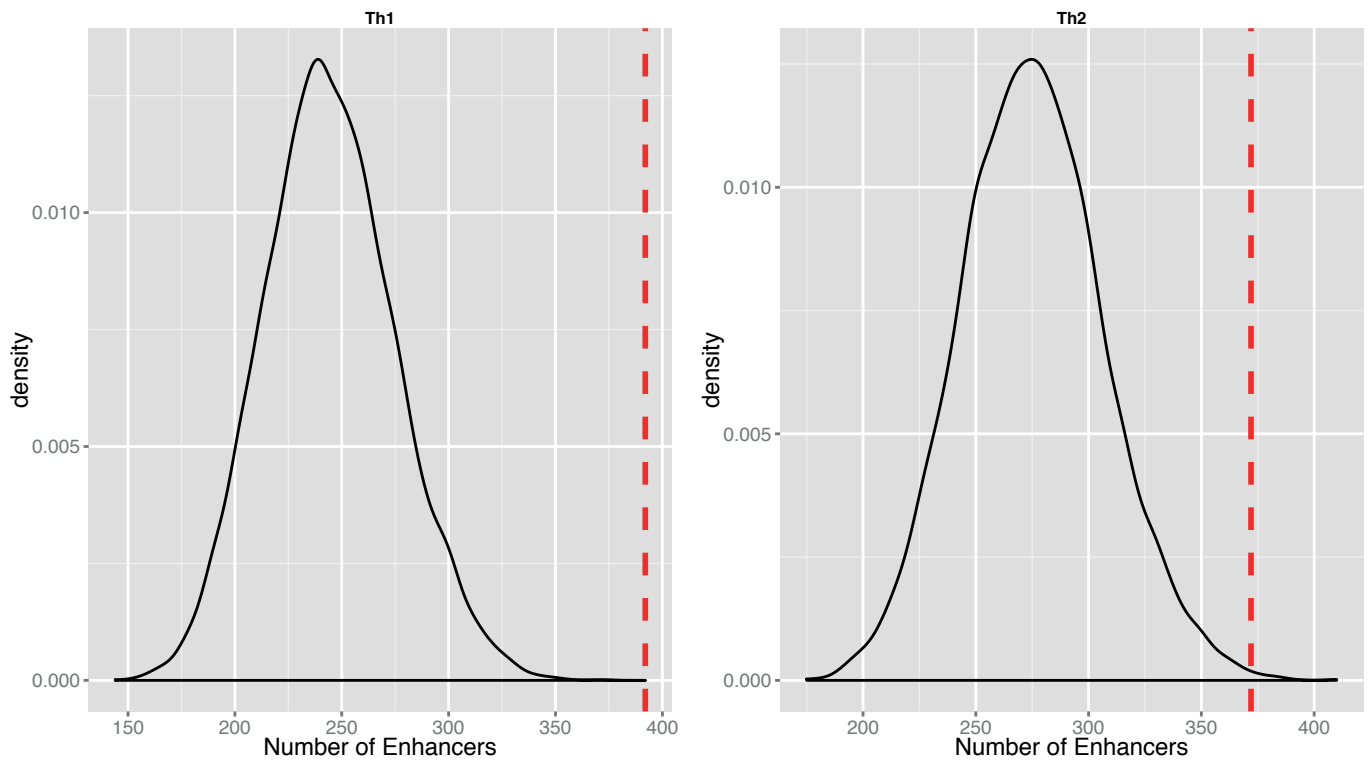

B

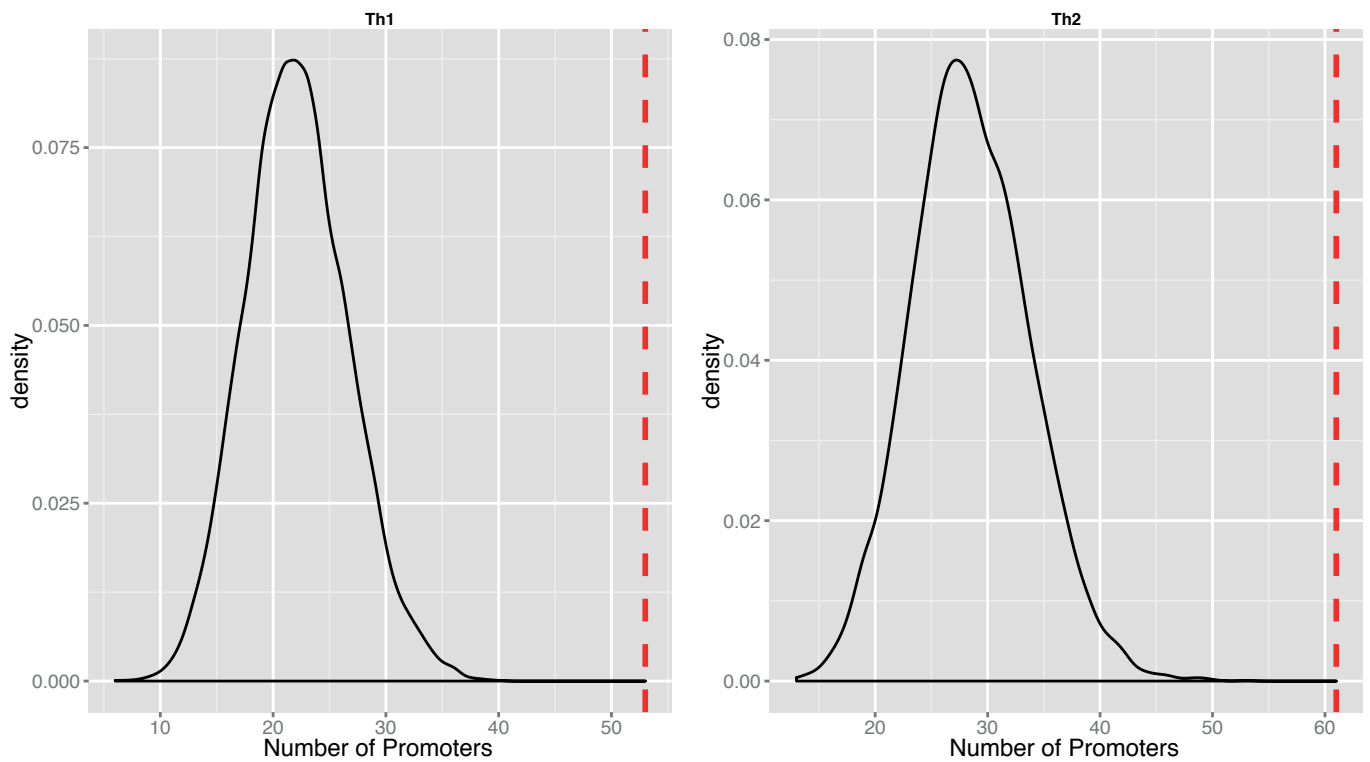

Supplement: Additional file 12: Figure S5. — Randomization test of epigenetic marks around lineage-specific lncRNAs. a Randomization test reveals that the number of lineage-specific enhancers around lineage-specific lncRNAs is more than anywhere else in the genome. b Randomization test reveals that the number of lineage-specific promoters around the lineage-specific lncRNAs is more than anywhere else in the genome. The distribution of enhancers or promoters in the vicinity of genes in the genome was determined by randomly picking the same number of genes as in the lineage-specific set. The red dashed line indicates the number of lineage-specific enhancers or promoters found in the vicinity of lineage-specific lncRNAs. (PDF 114 kb) [file 13073_2015_237_MOESM12_ESM.pdf]
